# A Case of Esophageal Squamous Cell Carcinoma Detected After Peroral Endoscopic Myotomy in a Patient With Achalasia

**DOI:** 10.7759/cureus.71604

**Published:** 2024-10-16

**Authors:** Tomohiro Kamio, Shoichiro Hirata, Kenta Hamada, Masaya Iwamuro, Motoyuki Otsuka

**Affiliations:** 1 Department of Gastroenterology and Hepatology, Okayama University Graduate School of Medicine, Dentistry, and Pharmaceutical Sciences, Okayama, JPN

**Keywords:** achalasia, esophageal cancer, esophagogastroduodenoscopy, peroral endoscopic myotomy, squamous cell carcinoma

## Abstract

Achalasia, a chronic esophageal motility disorder, increases the risk of esophageal squamous cell carcinoma. Despite effective treatment with peroral endoscopic myotomy (POEM), patients remain at risk of malignancy. We present the case of a 75-year-old Japanese woman diagnosed with achalasia who was found to have esophageal squamous cell carcinoma three months after POEM. Early detection through endoscopic surveillance and biopsy led to successful endoscopic submucosal dissection. This case underscores the need for ongoing surveillance of patients with achalasia post-POEM to ensure early identification and treatment of potential malignancies.

## Introduction

Achalasia is a chronic esophageal motility disorder characterized by impaired relaxation of the lower esophageal sphincter and loss of peristalsis in the esophageal body [1,2]. This often leads to dysphagia, regurgitation, and weight loss. Peroral endoscopic myotomy (POEM) is a minimally invasive surgical technique that has gained recognition as a promising treatment for achalasia, offering substantial symptom relief and improved patient outcomes [3,4]. Previous studies have demonstrated that POEM is a safe and highly effective treatment for achalasia in patients aged 65 and older [5,6]. Consequently, POEM is now regarded as the preferred approach for myotomy in this patient population.

Despite the effectiveness of POEM in managing achalasia, the risk of undiagnosed esophageal malignancies remains [7-10]. Esophageal squamous cell carcinoma is a relatively rare complication of chronic achalasia. The presence of esophageal cancer in patients with achalasia can complicate the diagnosis and treatment because the malignancy may be masked by underlying achalasia-induced esophageal mucosal inflammation or may become evident only post-treatment.

This report highlights a unique case in which a diagnosis of esophageal cancer was established following POEM in a patient with achalasia. The initial presentation, diagnostic workup, and subsequent findings underscore the importance of ongoing surveillance and careful evaluation of patients with achalasia even after successful intervention.

## Case presentation

A 75-year-old Japanese woman experienced gastroesophageal reflux disease symptoms for several years. Approximately one year previously, she occasionally noticed a sensation of food sticking in her throat. Her symptoms worsened two weeks prior to presentation, leading to difficulty eating and a weight loss of approximately 5 kg. She visited a local clinic, where esophagogastroduodenoscopy suggested achalasia. She was referred to a general hospital and admitted. On admission, she was unable to drink water and had mild pneumonia, likely owing to aspiration. Treatments included nil per os, intravenous fluids, and antibiotics. CT performed during hospitalization also revealed esophageal dilation and retention of residual material, consistent with achalasia. The patient was subsequently transferred to our hospital for treatment.

The patient had no history of alcohol consumption or smoking. She had been taking losartan and hydrochlorothiazide for hypertension and etizolam, brotizolam, and alprazolam for depression. Additionally, she had been taking a combination of ergotamine tartrate and anhydrous caffeine for migraines and vonoprazan and ecabet sodium for gastroesophageal reflux symptoms. Upon transfer to our hospital, laboratory tests revealed an elevated white blood cell count of 9,310/μL, a C-reactive protein level of 3.27 mg/dL, and anemia with a hemoglobin level of 10.2 g/dL (Table 1). Additionally, the albumin level was low at 3.5 g/dL. The squamous cell carcinoma-related antigen level was elevated at 4.56 ng/mL. Other biochemical parameters were within the normal ranges.

**Table 1:** Blood test results

Blood test results (units)	Patient value	Reference range
White blood cells (/μL)	9,310	3,300–8,600
Neutrophil (%)	80.3	40–70
Lymphocyte (%)	14.9	16.5–49.5
Monocyte (%)	4.2	2–10
Red blood cells (/μL)	3,470,000	4,350,000–5,550,000
Hemoglobin (g/dL)	10.2	13.7–16.8
Hematocrit (%)	31.8	40.7–50.1
Platelets (/μL)	263,000	158,000–348,000
Total protein (g/dL)	7.4	6.6–8.1
Albumin (g/dL)	3.5	4.1–5.1
Creatinine (mg/dL)	0.98	0.65–1.07
Sodium (mmol/L)	139	138–145
Potassium (mmol/L)	4.1	3.6–4.8
Total bilirubin (mg/dL)	0.44	0.4–1.5
Direct bilirubin (mg/dL)	0.15	0.08–0.28
Aspartate aminotransferase (U/L)	16	13–30
Alanine aminotransferase (U/L)	17	10–42
γ-Glutamyl transpeptidase (U/L)	21	38–113
Lactate dehydrogenase (U/L)	200	124–222
Alkaline phosphatase (U/L)	85	38–113
C-reactive protein (mg/dL)	3.27	0–0.15
Squamous cell carcinoma-associated antigen (ng/mL)	4.56	0.24–2.52

**Figure 1:**
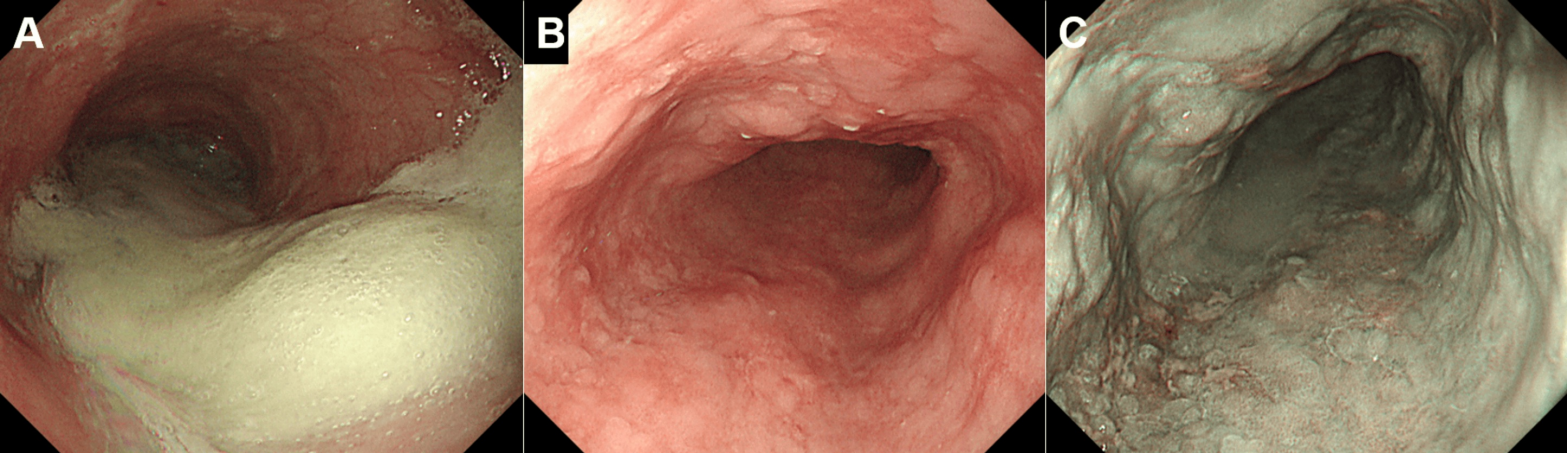
Endoscopic images prior to POEM Significant dilation of the esophageal lumen with retained mucus and saliva is observed (A). The esophageal mucosa appears diffusely coarse, with multiple areas of redness and shallow erosions (B: white light; C: narrow-band imaging). Endoscopic biopsy did not reveal squamous cell carcinoma.

Endoscopic examination revealed significant dilation of the esophageal lumen with retained mucus and saliva (Figure 1A). Resistance was encountered when the scope was passed through the lower esophagus. The esophageal mucosa was diffusely coarse with multiple areas of redness and shallow erosion but no obvious neoplastic changes (Figures 1B, 1C).

**Figure 2:**
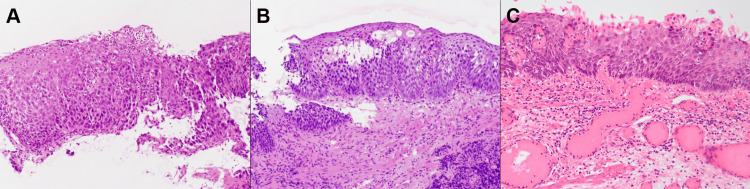
Pathological images An endoscopic biopsy taken from the esophagus prior to POEM revealed infiltration of inflammatory cells, including neutrophils, in the epithelium and lamina propria (A). The basal layer exhibited nuclear enlargement and increased cellularity, indicative of inflammatory changes. Endoscopic biopsy performed three months after POEM revealed squamous cell carcinoma (B). The resected specimen obtained via endoscopic mucosal dissection confirmed squamous cell carcinoma confined to the epithelial layer (C).

**Figure 3:**
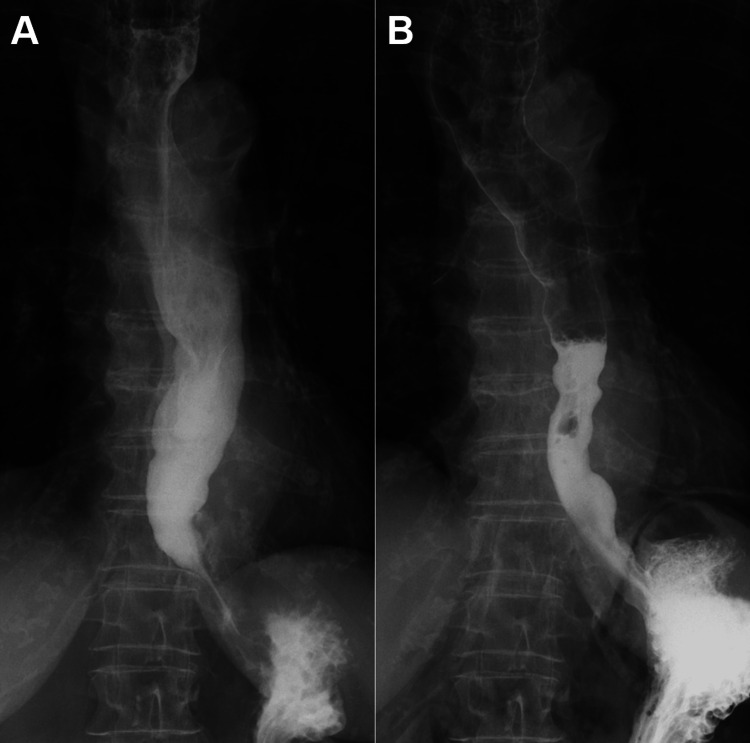
Contrast images before and after POEM The pre-POEM contrast study demonstrates bird’s beak-like narrowing, esophageal dilation with retention of contrast medium, and absence of peristalsis (A). The contrast study conducted the day after POEM shows partial improvement in the passage of contrast medium, with smooth flow from the esophagus into the stomach (B).

Biopsies were obtained from three sites in the esophagus, and pathological examination revealed non-neoplastic tissue with infiltration of inflammatory cells, including neutrophils, in the epithelium and lamina propria (Figure 2A). The basal layer showed nuclear enlargement and increased cellularity, indicating inflammation. Contrast imaging demonstrated bird’s beak-like narrowing, esophageal dilation with retention of contrast medium, and absence of peristalsis (Figure 3A). Based on these findings, the patient was diagnosed with esophageal achalasia, and POEM was performed. A contrast study conducted the day following POEM revealed partial improvement in the passage of the contrast medium, with smooth flow from the esophagus into the stomach (Figure 3B). The Eckardt score improved from 9 to 1 after POEM (Table 2) [11]. Although the level of squamous cell carcinoma-related antigen had been above normal prior to POEM, it normalized to 2.52 ng/mL following the procedure.

**Table 2:** The Eckardt score before and after peroral endoscopic myotomy (POEM)

	Prior to POEM	After POEM
Weight loss	2	0
Dysphagia	3	0
Retrosternal pain	1	1
Regurgitation	3	0
Total	9	1

**Figure 4:**
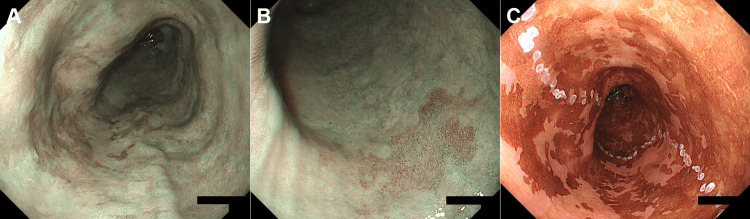
Images of the esophagus after POEM Narrow-band imaging identified brownish areas (A and B) where the biopsy confirmed squamous cell carcinoma. Iodine staining during endoscopic submucosal dissection highlights the esophageal carcinoma lesion as an unstained area surrounded by multiple faintly stained regions (C).

**Figure 5:**
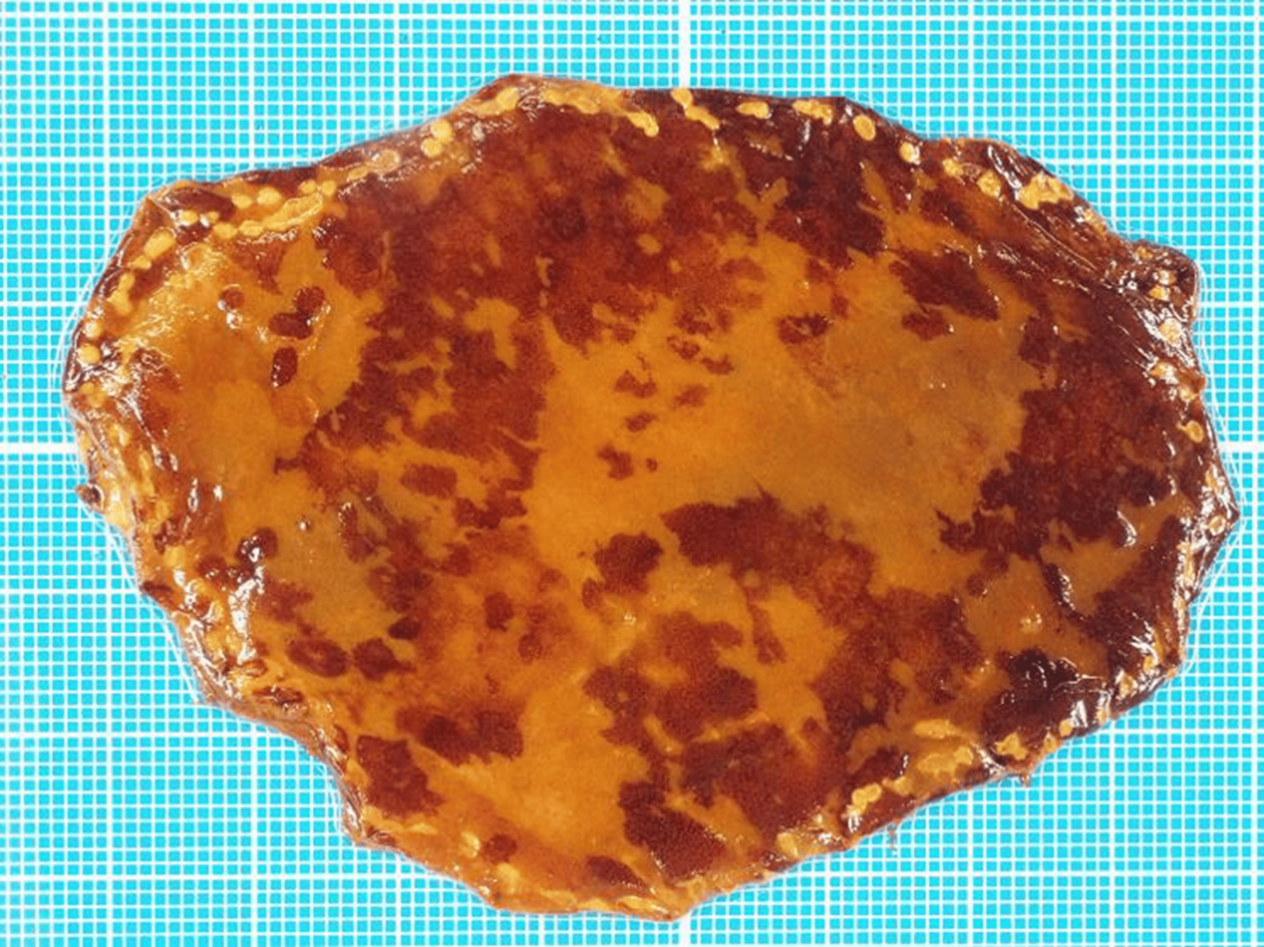
An image of the resected specimen The specimen shows multiple areas of dysplastic epithelium corresponding to squamous intraepithelial neoplasia surrounding the carcinoma lesion.

Three months after POEM, esophagogastroduodenoscopy revealed that although the esophagus remained dilated, residual material had not accumulated. The scope easily passed through the lower esophagus without resistance, and signs of reflux esophagitis were absent. Narrow-band imaging identified brownish areas located 26-32 cm away from the incisors (Figures 4A, 4B). Biopsy revealed squamous cell carcinoma (Figure 2B). We considered the lesion to be a type 0-IIb esophageal carcinoma and performed endoscopic submucosal dissection. Iodine staining revealed an esophageal carcinoma lesion as an unstained area surrounded by multiple faintly stained regions (Figure 4C). The resected specimen measured 70 × 48 mm and was diagnosed as pT1a-EP, pHM0, pVM0, Ly0, and V0, indicating curative resection (Figure 2C). Multiple areas of dysplastic epithelium corresponding to squamous intraepithelial neoplasia were also observed around the carcinoma lesion (Figure 5).

## Discussion

Achalasia is associated with an increased risk of esophageal cancer. Previous studies have shown that patients with achalasia have a 28-fold higher risk of developing esophageal cancer than the general population [12]. The prevalence of esophageal squamous cell carcinoma in patients with achalasia is 26-33 per 1,000 cases, whereas that of esophageal adenocarcinoma is 4 per 1,000 [13,14]. Despite the increased relative risk, the absolute risk remains low.

Given that chronic irritation and inflammation caused by achalasia can contribute to the malignant transformation of the esophageal mucosa, treatment for achalasia is believed to reduce the risk of developing esophageal cancer. However, this risk does not completely disappear [15], as high postsurgical incidences of esophageal cancer have been reported in patients with achalasia. In contrast, Ota et al. reported that 6 of 32 patients (18.8%) who underwent endoscopic surveillance following surgery developed esophageal cancer, suggesting a substantial risk even after chronic inflammation of the esophageal mucosa has been addressed [16]. Esophageal cancer has been reported 17-21.5 years after achalasia onset [17]. In the aforementioned study by Ota et al., esophageal cancer developed at an average of 14.3 years (range, 5-40 years) after surgery for achalasia [16]. Therefore, owing to the substantial risk, patients with achalasia may benefit from long-term endoscopic surveillance and maintain a high index of suspicion for esophageal cancer even after successful treatment [18].

In our case, the esophageal cancer was diagnosed 3 months following POEM. In this instance, the cancer was believed to be present prior to treatment, not newly developed after POEM, remaining undetected and masked both endoscopically and pathologically. Li et al. also reported a case in which esophageal cancer was diagnosed one year after POEM [19]. In situations where no interventions, such as POEM or surgery, have been performed, the accumulation of food debris, saliva, and mucus, along with their adherence to the esophageal mucosa, can hinder detailed observation. Furthermore, as in our case, inflammation may mask signs of cancer. Therefore, it is crucial to note that diagnosing coexisting cancer can be challenging in the presence of achalasia. In cases of significant esophageal mucosal inflammation, early endoscopic examination after POEM may facilitate the early diagnosis of concurrent neoplasms.

## Conclusions

This case report contributes to a growing body of evidence highlighting the association between achalasia and esophageal cancer. The challenge lies in differentiating cancer from inflammatory changes owing to achalasia, because the initial findings of esophageal dilation, retained mucus, and inflammation may mask an underlying malignancy. This highlights the importance of endoscopic reevaluation after POEM, particularly in cases of significant esophageal mucosal inflammation.
